# **3D-cell phantom-experimental setup to assess thermal effects and cell viability of lung tumor cells after electroporation**

**DOI:** 10.1038/s41598-024-78339-w

**Published:** 2024-11-07

**Authors:** Noah Müller, Severin Gylstorff, Heike Walles, Thomas Gerlach, Othmar Belker, Alessandro Zanasi, Daniel Punzet, Sascha Kopp

**Affiliations:** 1 Core Facility Tissue Engineering, Otto-von-Guericke University Magdeburg, Pfälzerstr.2, 39106 Magdeburg, Germany; 2 Experimental Radiology, University Clinic for Radiology and Nuclear Medicine, University Hospital Magdeburg, Leipziger Straße 44, 39120 Magdeburg, Germany; 3 Research Campus STIMULATE, Otto-von-Guericke University Magdeburg, Otto-Hahn-Straße 2, 39106 Magdeburg, Germany; 4 Department of Diagnostic and Interventional Radiology, Hannover Medical School, Carl-Neuberg-Straße 1, 30625 Hannover, Germany; 5 IGEA Zwgn. Deutschland, Feilitzschstraße 1, 80802 Munich, Germany; 6 Clinical Biophysics Laboratory, IGEA S.p.A, Via Parmenide 10a, Carpi, Italy

**Keywords:** Regenerative medicine, Cancer, Experimental models of disease, Translational research, Mechanical engineering, Cancer

## Abstract

Medical devices and technologies must undergo extensive testing and validation before being certified for public healthcare use, especially in oncology where a high research focus is on new advancements. Human 3D-tissue models can offer valuable insights into cancer behavior and treatment efficacy. This study developed a cell phantom setup using a rattail collagen-based hydrogel to facilitate reproducible investigations into ablation techniques, focusing on electroporation (EP) for lung tumor cells. The temperature rise due to the treatment is a critical aspect based on other studies that have discovered non-neglectable temperature values. A realistic physiological, biological phantom is crucial for electrode material development, non-thermal ablation control, tumor cell behavior study, and image-guided treatment simulation. The test system comprises a standardized 3D-printed setup, a cell-mimicking hydrogel model cultivated with NIH3T3 and HCC-827 cell lines. The treatment is evaluated with an AlamarBlue assay and the temperature is monitored with a sensor and a non-invasive MR-thermometry. Results showed the reliability of the selected monitoring methods and especially the temperature monitoring displayed interesting insights. The thermal effect due to EP cannot be neglected and it has to be discussed if this technique is non-thermal. The lesions in the phantom were able to show apoptotic and necrotic regions. The EP further led to a change in viability. These results suggest that the phantom can mimic the response of soft tissue and is a useful tool for studying cellular response and damage caused by EP or other treatment techniques.

## Introduction

Electroporation (EP) is a versatile minimal invasive technique utilized in different clinical and scientific fields like oncology, gene therapy, or microbiology and is described as non thermal^1^. The electroporation process involves the application of an external electric field with direct current (DC) to cells or tissues, creating micropores within the cell membrane. It can be used to introduce DNA molecules into cells, for electrochemotherapy due to the introduction of cytostatic drugs or as an ablation technique in the form of irreversible electroporation (IRE). The formation of pores in the cell membrane during electroporation occurs due to several mechanisms, including electrostatic charging of the lipid bilayer, rearrangement of the membrane proteins and mechanical stress on the membrane caused by the electric field^2^. These pores can be reversible or irreversible depending on the size^3^. Pores that do not reverse, initiate apoptosis in cells which is induced by the disruptive effect on cellular integrity and homeostasis. The process depends highly on the applied electric field, pulse parameters and the tissue of interest^1,2^. Studies evaluating the effect of different EP parameters have shown that the prediction of pore formation and cell viability based solely on given parameters (amplitude, duration, frequency and number of cycles) is unreliable, due to different tissue types and electric field distribution^4^. The applied voltage was shown to solely be a linear predictor.

As result, the need for biomaterial-based 3D tissue models, enabling the research on cellular responses to EP, is high. These models, following the 3R-principle (Refine-Reduce-Replace), are more time- and cost-effective, allowing for standardized high throughput analysis while representing very close proximity to human physiology^5,6^. Indeed, the Food and Drug Administration (FDA) stated that “it is important to recognize that considerably more research and development is needed for tools that might replace, reduce, or refine the large battery of animal studies [...]”^7^. To investigate EP, several in-vitro models are available. Biological in-vitro models have shown promising capabilities to investigate EP ablation efficacy and the evaluation of cell responses with different stainings regarding^8,9^. However, these models lack crucial aspects for a holistic evaluation like the applied temperature, the influence of electrode material and long-term cell responses. A systematic review by Hogenes et al.^4^ showed that the quality of studies investigating the effect of electroporation was mainly low for 14 out of 18 studies (78 %). The most common are 2D and 3D cell cultures and vegetable models. Additionally, 3D spheroid models have been developed and utilized for studying EP. These models offer advantages such as a closer approximation to the tumor microenvironment and the ability to study cellular responses in a 3D context. However, they also have limitations, such as challenges in replicating larger tissue volumes and variations in spheroid size and shape, which can affect the consistency of results^10^.

Our model aims to address these limitations by enabling the study of larger volumes and offering more transferable results in certain aspects. Therefore, a standardized experimental setup combined with methods to monitor and evaluate is presented in this study to understand the effect of EP as well as the influence of specific parameters and treatment protocols.

## Methods

### Cell culture

Mouse fibroblasts NIH3T3 (American Type Cultur Collection, Virginia, USA, CRL-1658) were cultivated in DMEM high glucose (Sigma-Aldrich, Taufkirchen, Germany, D5796) supplemented with 10 % FCS (Bio&Sell, Nürnberg, Germany, FBS.S0615). Human adenocarcinoma cells HCC-827 purchased from the DSMZ (no. ACC 566) were cultivated in RPMI1640 (Thermo Fisher Scientific Inc., United States, 61870010) supplemented with 20 % FCS (Bio&Sell, Nürnberg, Germany, FBS.S0615). Both cell lines were cultivated under standard cell cultures conditions (37 °C, 5 % CO2).

### Cell-laden phantom

Hydrogel models were composed of 2/3 rattail collagen-1 (10,5 mg/ml) (Fraunhofer-Institut, Germany) and 1/3 gel neutralization liquid (GNL) (Fraunhofer-Institut, Germany). In preparation of the cell-laden phantoms, 10^6^ cells/ml were added into GNL before mixing both components over a 3-way-valve to initiate polymerization (Discofix C by B. Braun, Germany). Gels were cultivated in an insert (ThinCert by Greiner BioOne, Germany) placed into a 12-well plate (VWR, Belgium) and supplemented with cell-specific medium respectively.

### Gelatine phantom

Gelatine phantoms were produced by mixing 29 mg/ml gelatine powder (Dr. Oetker, Germany), 10 mg/ml collagen powder (Vit4ever, Germany) and 30 % DMEM medium (Sigma-Aldrich, Taufkirchen, Germany, D5796) on a heating plate before it got poured into a container and placed at 4 °C.

### 3D printing

All 3D-printed parts were modelled using Fusion 360 (Autodesk Inc., United States, 2.0.18961), followed by exporting STL files and slicing using Ultimaker Cura 5.2.1 (Ultimaker B.V., Netherlands). An SV06 fused deposition modelling (FDM) 3d printer (Sovol 3D, China), facilitating a 0.4 mm nozzle was used to manufacture the parts from polypropylene filament (PP natur by Fiberlogy, Poland). The printing temperature was set at 235 °C and the bed temperature was 80 °C. Due to adhesion difficulties, the printer bed was coated with the Magigoo Pro PP (Thought3D Ltd, Malta). Sterilization took place at 121 °C in a LABOKLAV 135 MS autoclave (SHP Steriltechnik AG, Germany). The biocompatibility was tested following the guidelines for in vitro cytotoxicity and sample material defined in ISO-10993-5 and ISO-10993-12. Autoclaved PP cubes (6 mm x 6 mm x 3 mm) with the specific size and surface to achieve 3 cm^2^/mL were used to perform a direct and indirect test over 24 h. A MTT following a manufacturers protocol (Sigma-Aldrich, Taufkirchen, Germany, M2128-1G) was performed to measure the relative cell viability (RCV).

### Electroporation devices

Two different devices were used to apply the electric pulses for electroporation. The Genedrive by IGEA SpA. (Carpi, Italy), which was specifically developed for preclinical studies and allows for a customization of the key electrical parameters (number, amplitude, frequency, duration and type of pulses) and an experimental MR-conditional EP device developed at STIMULATE campus^11^. The same plate electrodes were used with both devices (Customized Adjustable Plate Electrode, IGEA).

### EP-procedure

Hydrogels were cultivated for 24 h before EP. The hydrogels were treated with electroporation buffer^12^(10 mM HEPES, 250 mM sucrose, 0.7 mM MgCl2, 0.3 mM CaCl2) to stabilize the electrical conductivity during the ablation. Gels were washed 3x times with PBS^−^ followed by incubation for 10 min with 100 µl electroporation buffer. Both devices were set to deliver 8 rectangular pulses with 1000 V/cm, 160 µs pulse length and 25 cycles. The STIMULATE device had a pause time of 1000 ms between the cycles, while with the IGEA Genedrive, every cycle is started manually due to device safety. This results in a overall expanded treatment time with the IGEA device. Per cell line *N* = 8 hydrogels got treated while *N* = 8 untreated controls were performed. An AlamarBlue assay was done 24 h after the ablation. Histological sectioning was done 48 h after ablation (see section “Analyses”).

### Monitoring

#### Temperature

The temperature was monitored during the treatment with a fiber optic temperature sensor (Optocon TS5/A366 by Weidemann Technologies, Germany) and a multi-channel thermometer (FOTEMP4-16 by Weidemann Technologies, Germany), which can be accessed via a laptop.

#### Oscilloscope

The STIMULATE device has no interface showing the delivered pulses. Visualisation was achieved using a Rigol DS1054Z Digital-Oscilloscope (Rigol Technologies, Germany) which monitors the delivered voltage and current of the pulses.

#### Proton resonance frequency shift thermometry

Proton Resonance Frequency Shift Thermometry (PRFS-Thermometry) is a non-invasive imaging technique that provides temperature change maps with high spatial resolution. PRFS-Thermometry relies on the resonance frequency of hydrogen atoms being approximately linearly dependent on temperature in molecules with hydrogen bond-bound hydrogen at temperatures around room temperature. By determining the phase difference between post- and pre-ablative in MR image phases, the temperature change can be calculated by the following mathematical model^13^:

1 $$\:\varDelta\:\text{T}=\:\frac{{{\upphi\:}}_{\text{p}\text{o}\text{s}\text{t}-\text{a}\text{b}\text{l}\text{a}\text{t}\text{i}\text{o}\text{n}}\:-\:{{\upphi\:}}_{\text{b}\text{a}\text{s}\text{e}\text{l}\text{i}\text{n}\text{e}}}{\gamma\:\alpha\:{B}_{0}TE}$$

$\:\gamma\:$- gyromagnetic ratio of hydrogen; $\:\alpha\:$- constant of correlation; $\:{B}_{0}$ - magnetic field strength; $\:TE$ - echo time.

Images were acquired on a 3T Siemens Magnetom Skyra using a Cartesian gradient recalled echo sequence (GRE) with a field of view (FoV) of 180 × 180 mm^2^, slice thickness 3 mm, resolution in plane 0.7 mm, TR 50 ms, TE 5 ms, flip angle 15 deg.

The baseline and post-ablative acquisition durations were approximately 1:17 min each (6 Averages). Inter-ablative thermometry was not feasible in the used setup due to electromagnetic interference.

Retrospective image analysis was performed using MATLAB [9.10.0 (R2021a)]. The acquired phase data was converted into radians, and temperature change maps were calculated using Eq. (1). A temperature offset was determined from a water bottle positioned at the edge of the FoV.

### Analysis

#### Assays

**Fig. 1:**
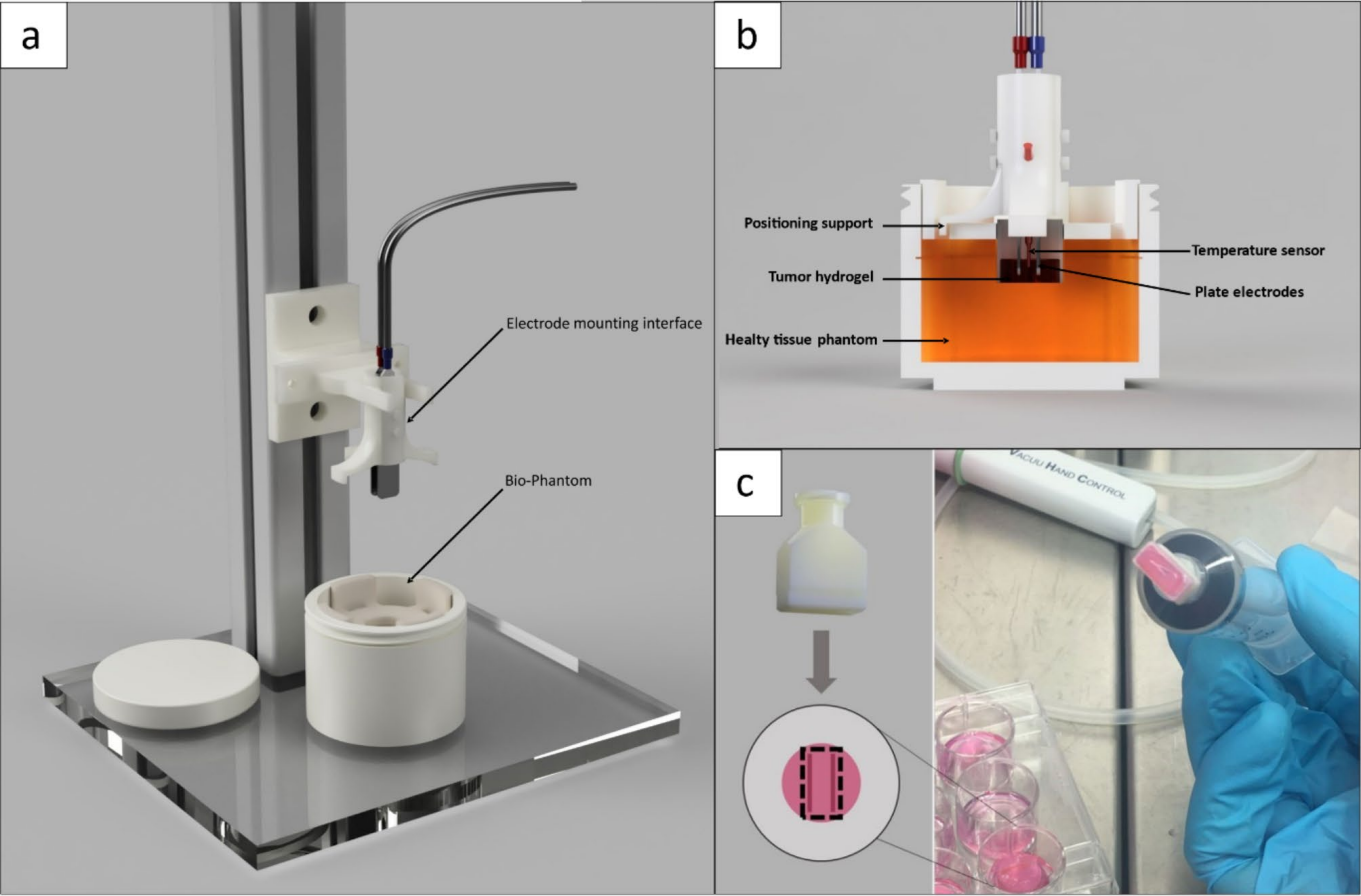
**A**: Experimental setup for an EP procedure including the electrode mounting interface positioned on a rail and the Bio-Phantom;** B**: Cross-section of the electrode mounting interface including the positioning support, temperature sensor and plate electrodes, and the Bio-Phantom consisting of the tumor hydrogel, healthy tissue phantom **C**: 3D-printed biopsy punch for isolating the ablation area of a hydrogel. The printed part is connected with a luer-lock system to a 20 ml syringe.

The AlamarBlue assay was performed following the standardized manufacturer protocol (Thermo Fisher Scientific Inc., United States, DAL1025). The incubation time was set to 20 h as a result of the standard curve. With respect to measuring the metabolic activity in the intended ablation area, located between the electrodes, a rectangular-shaped 3D-printed vacuum biopsy punch was used (See Fig. 1C). Cell medium was used without adding FBS due to the possibility of mitigating the metabolism of the resazurin^14^. The fluorescence intensity was measured at 560 λ excitation and 590 λ emission using an Infinite 200 Pro microplate reader (Tecan Group Ltd., Switzerland). The percentage difference between treatment and control was calculated following Eq. (2):

2 $$\:\text{\%}-\text{d}\text{i}\text{f}\text{f}\text{e}\text{r}\text{e}\text{n}\text{c}\text{e}\:\text{b}\text{e}\text{t}\text{w}\text{e}\text{e}\text{n}\:\text{t}\text{r}\text{e}\text{a}\text{t}\text{m}\text{e}\text{n}\text{t}\:\text{a}\text{n}\text{d}\:\text{c}\text{o}\text{n}\text{t}\text{r}\text{o}\text{l}=\frac{\text{T}\text{r}\text{e}\text{a}\text{t}\text{e}\text{d}\:\text{s}\text{a}\text{m}\text{p}\text{l}\text{e}\:-\:\text{B}\text{l}\text{a}\text{n}\text{k}}{\text{U}\text{n}\text{t}\text{r}\text{e}\text{a}\text{t}\text{e}\text{d}\:\text{s}\text{a}\text{m}\text{p}\text{l}\text{e}\:-\:\text{B}\text{l}\text{a}\text{n}\text{k}\:}\times\:100$$

#### Histological analysis

After treatment, samples were embedded in Histofix − 4 % formaldehyde (ITW Reagents, United States) overnight at 4 °C, followed by histological section cutting. Dehydration of samples was achieved using an ascending alcohol series (Isopropanol 20 − 100 %) for 24 h using an automated tissue processor (Leica Biosystems, Germany). Afterwards paraffin embedding was performed with a Histostar embedding station (Thermo Fisher Scientific Inc., United States). Precise cutting was executed using a rotary microtome HM355S (Thermo Fisher Scientific Inc., United States) with 10 μm slice thickness. The hematoxylin and eosin, as well as the alcian blue staining, are following standardized protocols^15^.

## Results

### Experimental setup

The experimental setup for the EP follows a modular concept with several exchangeable 3D-printed parts (Fig. 1). All components can be sterilized and placed under a biosafety cabinet to achieve sterile working conditions. Figure 1A shows the experimental setup, including the electrode mounting interface fixed onto a rail and the Bio-Phantom positioned underneath it. The electrode mounting interface (See Fig. 1A-B) can be equipped with different electrode distance spacers and is modifiable to mount several electrodes or comparable ablation devices. Figure 1B illustrates the cable duct which allows the fiber optic sensor to access the ablation area, while a positioning support ensures a parallel placement of the electrode with a fixed penetration depth. The insert, in concert with the hydrogel, is placed into a mount in the pocket of the healthy tissue phantom. The mold is created by placing a PP cylinder during the gelation process and removing it afterwards. While the cell-laden hydrogel measures a volume of 1 cm^3^, the healthy tissue phantom has a size of 60 cm^3^. The ablation device was precisely inserted through vertical movement on the rail into the phantom placed in cutout of the glass bottom. A rectangular vacuum biopsy punch, displayed in Fig. 1C, was developed to separate the intended ablation area, while preventing the surrounding tissue affecting the metabolic assay.

**Fig. 2:**
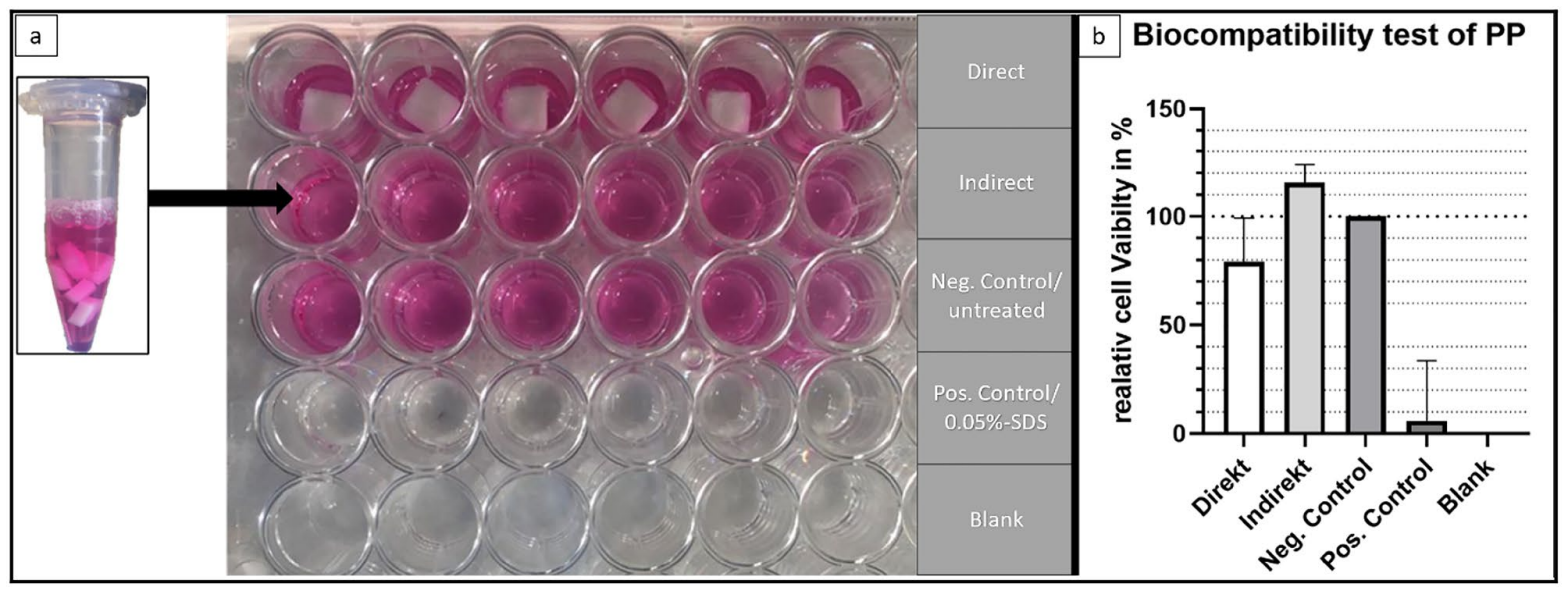
Experimental setup of biocompatibility test (ISO 10993-5) with 5 × 10^4 ^NIH3T3 cells per well. (**A**) Presents the MTT assay setup with direct contact of sterilized 3D-printed PP cubes in the first row. The indirect assay, in the second row, was executed with cell medium which was incubated for 24 h with sterilized 3D-printed cubes. Negative control is untreated, positive control was incubated with 0.05 % SDS and the blank stays empty with no cells seeded. The Well-plate was incubated for 24 h before measurements to allow cells to react to the treatments. (**B**) The relative cell viability (RCV) measured using the MTT assay is shown in the graph with the negative control set to 100 %.

The developed parts used in the setup, like the biopsy punch, have direct contact with cellular components, which makes the biocompatibility of the printing material necessary. The results and setup of the executed test is seen in Fig. 2A-B. The direct test with the autoclaved PP cube for *N* = 6 shown in row one (Fig. 2A) had a relative cell viability (RCV) of RCV_direct_ = 79 %. The indirect test with cell media incubated for 24 h with PP cubes shown in row two (Fig. 2A) had a RCV_indirect_ = 115 %. The negative control is untreated and is set to be the reference for 100 % cell viability.

### MRI and CT

Potential compatibility and reliability across diverse imaging applications allows versatile investigations and monitoring of the Bio-Phantom.

**Fig. 3:**
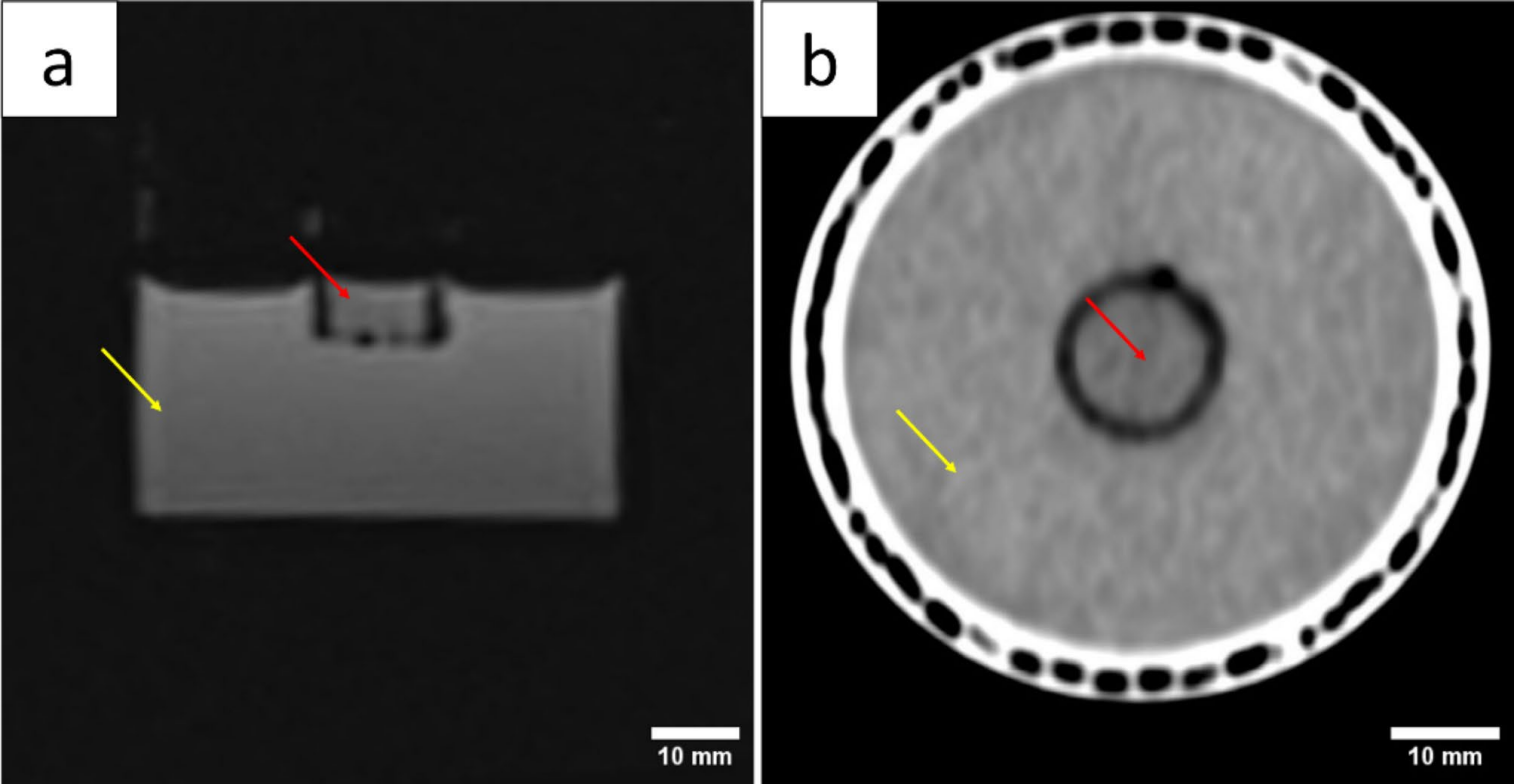
Bio-Phantom consisting of healthy tissue phantom (yellow arrow) and tumor hydrogel (red arrows) captured with **A**: MR imaging (3T Siemens Magnetom Skyra with a T1-vibe sequence) and **B**: CT (Siemens SOMATOM X.cite).

The MR image shown in Fig. 3A was acquired using a 3T Siemens Magnetom Skyra with a T1-vibe sequence (field of view (FoV) of 96 × 96 mm^2^, resolution 1 × 1 × 1 mm^2^, TR: 7.17 ms, TE 2.93 ms, flip angle: 12°). It shows a slice of the Bio-Phantom in the sagittal plane, where the cell-laden collagen hydrogel is marked with a red arrow, while the healthy tissue phantom by a yellow arrow. Both portions are displayed as a homogenic mass. Small artefacts are visible in the surroundings of the hydrogel, where the insert is placed.

The CT image was acquired using a Siemens SOMATOM X.cite with the following parameters: Collimation, 38.4 × 0.6 mm; Voltage, 70 kV; Tube current 28 mA; Slice thickness 2 mm; Pitch factor 0.8. The raw data was reconstructed using a Br40 convolution kernel. Figure 3B shows a slice of the Bio-Phantom in the transverse plane with the cell-laden hydrogel marked in red and the healthy tissue phantom in yellow, while the PP casing is visible on the outskirt. Cell-laden hydrogel as well as the surrounding matrix presented a homogenic mass.

### Temperature monitoring

To examine the impact of an EP, it is essential to monitor the temperature rise to ensure that cells are not at risk of undergoing thermal necrosis.

**Fig. 4:**
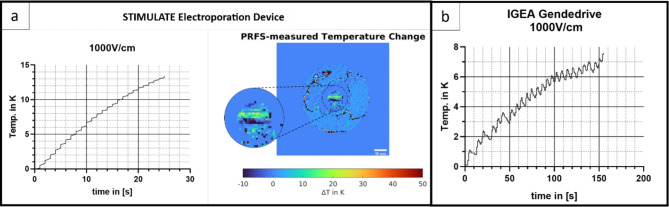
**A**: Temperature measured during treatment with the Stimulate IRE at E = 1000 V/cm with 25 cycles of 8 pulses (left) in the center of a hydrogel with a fiber optic temperature sensor (left) displaying ∆T = 14 K, ∆T/∆t ≈ 0.5 K/s, and PRFS-thermometry of Bio-Phantom(right) displaying ∆T ≈ 10–15 K in the ablation area. **B**: Temperature curve measured in the center of a hydrogel with a fiber optic temperature sensor during treatment with the IGEA GeneDrive at E = 1000 V/cm with 25 cycles of 8 pulses. The graph displays ∆T = 7.5 K ∆Tc ≈ 0.8 K/cycle.

The temperature monitoring with the fiber optic sensor in the center of the ablation area is exemplary shown for single measurement with 1000 V/cm in Fig. 4 displaying a temperature increase for both devices. The STIMULATE device led to a linear increase (R^2^ = 0.98) of ~ 13 K over a time span of 0:27 min resulting in ~ 0.5 K/s (Fig. 4A). While the IGEA Genedrive, with a longer treatment time of in total 2:35 min, led to an increase of ~ 8 °C with a linear coefficient of determination R^2^ = 0.95. The graph in Fig. 4B shows 25 peaks related to 25 induced pulse cycles.

In addition, non-invasive temperature monitoring was performed using MR PRFS-thermometry. Figure 4A shows the result after treatment with the STIMULATE device determining a temperature increase of approximately 10–15 K in the area between the electrodes. The IGEA device is not compatible in the MR environment, thus hindering the performance of a MR thermometry. Noise is shown throughout the thermometry image and artefact are displayed especially in gelatine phantom and the area around of the hydrogel.

### Analysis

All histological sections displayed were cut in the transverse plane of the hydrogel, separated by the rectangular biopsy punch.

**Fig. 5:**
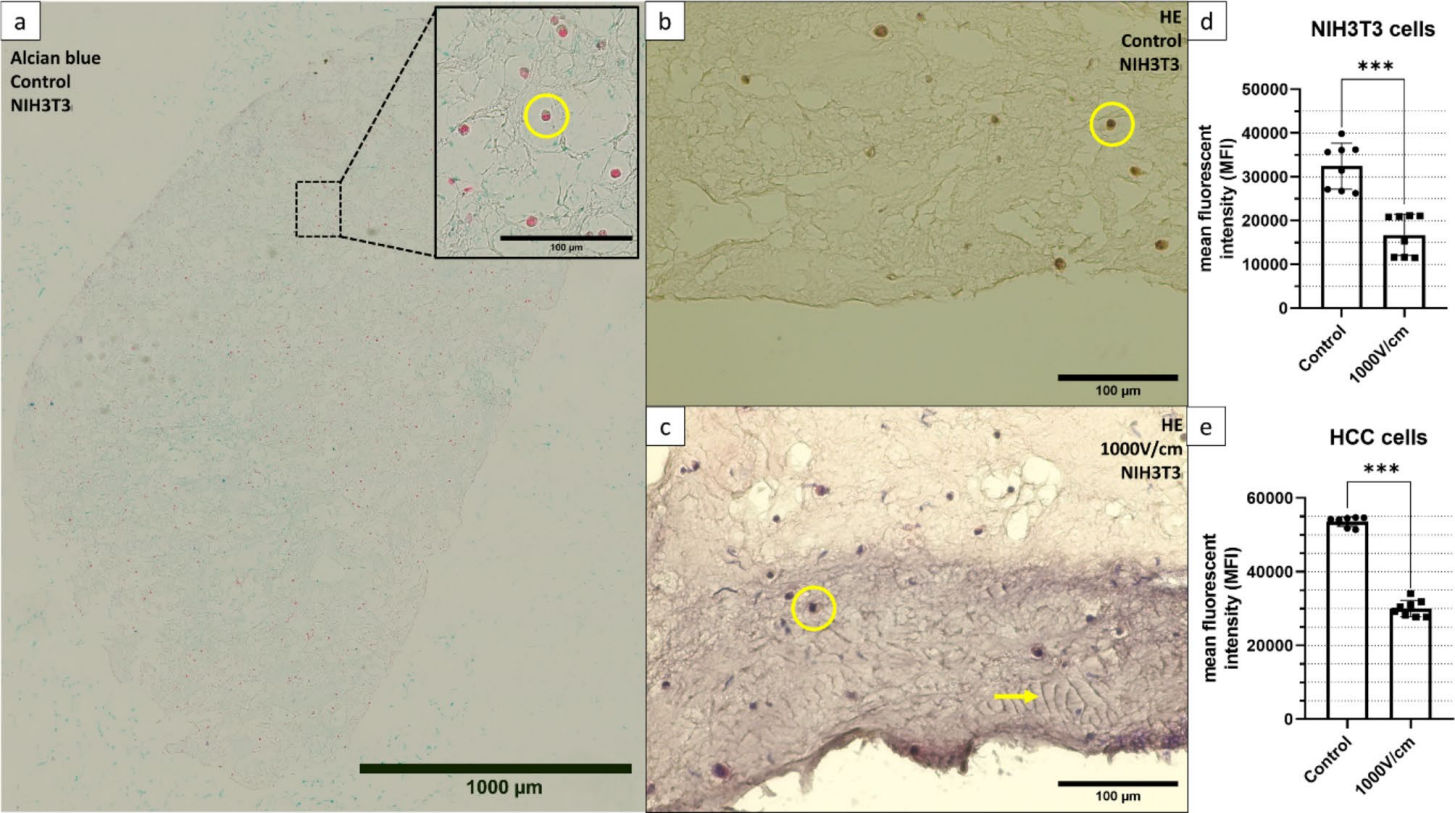
**A-C** Stainings of paraffin-embedded collagen-based hydrogel containing NIH3T3 cells (yellow circle). **A**: Alcian blue staining displaying the cell distribution throughout the section. **B**: HE staining of untreated control with homogenic staining of the ECM. **C**: HE staining of hydrogel treated with Stimulate IRE at E = 1000 V/cm with 25 cycles of 8 pulses, displaying a ~150µm wide purple-stained area at the insertion point of the plate electrode and fiber-like structures marked with a yellow arrow. **D-E**: Relative metabolic activity of [N=8] collagen-based hydrogels 24 hours after treatment with the Stimulate IRE at E = 1000 V/cm with 25 cycles of 8 pulses for **D**: Cultivated with 1 × 106 HCC-827 cells. **E**: Cultivated with 1 × 106 NIH3T3 cells. The reference were each N = 8 untreated hydrogels. Values are generated with an AlamarBlue assay with 20 hours of incubation time.

The alcian blue staining (Fig. 5A) of the control with NIH3T3 cells shows the cell distribution throughout the gel with a cell exemplary marked with a yellow circle. To investigate homogenic cell distribution the coefficient of variance of the mean near-neighbour distance (COV_d_) was calculated with Eq. (3)^16^

3 $$\:{COV}_{d}=\frac{{S}_{d}}{d}$$

s_d_ = standard deviation; d = mean nearest-neighbour distance.

In a study by Ayyar et al.^16^ the particle distribution was categorized as follows: ordered distribution COV_d_ = 0.09, random distribution COV_d_ = 0.32 and clustered distribution COV_d_ = 0.69. The histological section presented in Fig. 5A has a calculated value of COV_d_ = 0.49. Cell counting and distances were acquired using ImageJ.

In Fig. 5B the HE staining of the control displays the cytoplasm with dark purple stained cells marked exemplary with a yellow circle.

The HE staining of the sample treated by the STIMULATE device with 1000 V/cm, 8 pulses and 25 cycles is shown in Fig. 5C. In the area of the inserted electrode is, compared to the control in Fig. 5B, a section with a depth of ~ 150 μm in purple. Fiber-like structures occur throughout this segment marked with a yellow arrow. Figure 5D, E display the result of the fluorescence intensity measurements for the AlamarBlue assay of NIH3T3 and HCC-827 cells treated with the STIMULATE device. The metabolic activity of treated HCC-827 cells decreased by 44 % compared to the controls, whereas NIH3T3 cells exhibited a 49 % reduction. NIH3T3 cells have a higher standard deviation of σ_C_ = ± 17 % and σ_IRE_ = ± 15 %, while HCC-827 is at σ_C_ = ± 2 % and σ_IRE_ = ± 4 %. These results are significant with *p* < 0.0001 for 95 % confidence interval.

## Discussion

### Experimental setup

New medical devices and technologies have to be tested, optimized and validated before they can be certificated according to the medical device regulation (MDR) and transferred into the public health care system^18^.  In-vitro models are less time consuming and less expensive compared to animal models, allowing rapid prototyping. These models can be used to validate the devices but also can be used for research purposes^17^. In this case, human 3D tissue models have wide application options to help understand cancerous behaviour and evaluate cancer treatments. The rapid evolution of medical technologies and devices, coupled with the growing shift towards alternatives to animal models, like the Directive 2010/63/EU of the European Parliament which was implemented as “an important step towards achieving the final goal of full replacement of procedures on live animals for scientific and educational purposes”^19^, is increasing the demand for suitable models. Essential for creating such models is the combination of a Bio-Phantom, reliable treatment procedures and readout methods.

When establishing an experimental setup for the evaluation of EP, the standardized treatment and the monitoring of thermal impacts is crucial to ensure cells do not undergo thermal necrosis^20^. The electrode mounting interface developed in this study combines these requirements by ensuring a reliable electrode placement while being able to adjust the electrode distance and having an access point for the temperature sensor. It also ensures a parallel positioning of the electrodes which is necessary for an adequate EP, because an uneven electric field distribution can cause an inconsistent exposure and possibly lead to varying treatment results^21^. In previous studies, when collagen-based hydrogels were facilitated, no needle guidance was implemented^9^.

The setup is mostly composed of PP which needs to be biocompatible due to direct cell contact. The biocompatibility was tested using an MTT test (Fig. 2) resulting in a RCV above 70 % for the direct as well as the indirect contact to the material. With accordance to ISO 10993-5 standards, the material can therefore be considered as non-cytotoxic.

Histologic sections (Fig. 5A) of the hydrogel itself showed to have cell distribution in between a random and a clustered value. Clustering in 3D cell cultures is a typical phenomenon due to a non-uniform ECM^22^. The alcian blue staining (Fig. 5A) further showed no optical cell polarization is present throughout the section indicating a successful nutrient supply throughout the gel^23^.

### Imaging compatibility

Compatibility with imaging techniques allows for the utilization of non-invasive monitoring methods. In combination with devices that can be used in MR environments, like the STIMULATE device, the Bio-Phantom can be used for the training of image-guided interventions, followed by treatment analysis using multiple assays. Further imaging sequences can be optimized for specific ablation techniques. Another field of application is the Bio-Phantom as a training tool for physicians. A study from McHugh et al.^24^ demonstrated the ability of a cell mimicking phantom and the use of Apparent Diffusion Coefficient (ADC) MR imaging to detect tumor tissue. Adding spheroids into the hydrogels like in a study from Kim et al.^25^ could be used to train physicians to detect tumor sites.

The MR-image of the Bio-Phantom showed artifacts in the area around the hydrogel (Fig. 3A), which can be explained as susceptibility artifact due to air enclosures^26^. The separation of the hydrogel and the gelatine phantom by the plastic insert is the reason for that. To improve the image quality and avoid artifacts, the Bio-Phantom should be cultured as one hydrogel containing a healthy tissue and a tumor site, which could be realized with a spheroid^27^. Upscaling the hydrogel would further enhance its image quality and the size also limits potential MR techniques like the thermometry shown in Fig. 3A. The resolution is not ideal, due to parameters being optimized to detect temperature changes and not for anatomic images. A larger model would enable electrodes with more needles used for larger ablation area.

### Temperature

Electroporation is stated to be a non-thermal technique with the benefit of preserving the extracellular matrix (ECM), while creating pores in the cell membrane^1^. The associated temperature changes during the electroporation process remain a subject of debate and investigation^21^. The extent and implications of temperature rise during electroporation procedures can have profound effects on cell viability, membrane integrity, and overall treatment outcomes^21^. When using EP as an irreversible ablation technique for tumor cells, the aim is the disruption of the cell membrane integrity leading to apoptosis, rather than necrosis. Necrotic cell death leads to the release of inflammatory cellular contents and can, especially in tumor tissue, promote the effect of metastasis^28^.

The temperature rise of ~ 10–15 K associated to EP pulse protocols displayed by the temperature curves in Fig. 4 was comparable to other studies using tissue mimicking phantoms or simulations^4,9,29^. However, these studies included no cell-laden phantoms to correlate the thermal effect on cells and the ECM. Dewhirst et al.^30^ showed that the thermal necrosis process has an exponential correlation between temperature and the exposure time with 43 °C as a breakpoint. At 43 °C cells exhibited signs of thermal necrosis after ~ 500 min, whereas at 55 °C necrosis was initiated after 30 s. These results demonstrate the importance of monitoring the temperature and the influence of EP parameters (Field strength, Electrodes distance, pulse numbers, pause time) as well as the different electrodes. A study by van den Bos et al.^21^ demonstrated the correlation between temperature rise and increasing voltage, pulse length and electrodes distance. Comparing both devices used for this study the longer pause time between pulse intervals of the IGEA Genedrive showed the tissue to cool down ~ 0.5 K after every peak. This also showed that the developed experimental setup can help adjusting treatment planning to stay under the necrotic temperature thresholds. The H&E stainings in Fig. 5B-C display at the insertion point of the electrode plate anomalies that have similarities to a burn wound, while the alcian blue staining eliminates the possibility of a dye error. During a study by Cannon et al.^31^ H&E staining was performed to identify burn depth on the skin of a pig which presented comparable purple sections with an increasing area for longer burn time. The different dye gradients can occur due to collagen denaturation. Furthermore, in Fig. 5B bundled collagen fibers (yellow arrow) are present, which is an indicator of a thermal influence^32^. Another study^21^ revealed the incident of light flashes on the negative electrode during EP. Together with the rise of the temperature measured with the fiber optic sensor and the MR-thermometry a tissue burn can be assumed. Further assays, like the investigation of heat shock protein markers need to be performed to validate these findings on a cellular level.

It also seems feasible to simulate numerically. However, numerical simulations have inherent limitations, such as accurately modeling the complex biological responses and thermal effects observed in experiments. Correlating experimental results with simulations could provide deeper insights into the electroporation process, but it requires precise input parameters and validation against experimental data to ensure reliability and applicability. The integration of experimental and simulation approaches could enhance the understanding of EP effects, yet challenges in accurately replicating biological conditions and responses remain.

### Cell viability test

Cell viability tests measure the overall health, activity, and functionality of a cell and values can be correlated to estimate the number of living cells^33^. The AlamarBlue assay used in this study is based on the reduction of resazurin to highly fluorescent resorufin by metabolic active cells and is published to be non-cytotoxic at the working concentration of 10 %^34^. A study by Bonnier et al.^35^ covers a comparison of the use in 2D and 3D cell cultures. The result showed that the assay is reliable when prolonging the incubation time due to the diffusion time into the 3D matrix. In accordance, a suitable incubation time was measured at 20 h by creating a standard curve.

Other studies showed that the optimal timepoint for measuring the cell viability after an EP was 24 h post treatment^4^. This is due to the fact that cells can recover from certain membrane disruptions and there are differences in cell line-specific membrane repair mechanisms. A study^36^ has shown that tumor cells have strong repair mechanisms against membrane disruption due to ESCRT processes (Endosomal sorting complexes required for transport). This would lead to the assumption that the viability of tumor cells after the EP should be higher. Results displayed in Fig. 5C-D showed contrary values with HCC-827 cells having a lower viability then NIH3T3 cells. Tumor cells are in general less mechanical resistant and the induced mechanical stress due to the EP can be the consequence for that^37^.

The results of the AlamarBlue assay displayed in Fig. 5D-C indicates a cell viability reduction after EP of under 50 %. Since the measured region was exclusively the ablation area, separated by a biopsy punch, a higher decrease of the cell viability was expected since the field strength of E = 1000 V/cm is stated to lead to irreversible cell damage^5,38^. The thermal effect due to EP cannot be neglected and it has to be discussed if EP is a non-thermal technique. Further investigations on heat stress markers are need to be performed to trace back the cell viability reduction onto the pore formation or thermal necrosis.

## Conclusion

In conclusion, an experimental setup was developed to establish a reliable treatment process integrating a monitoring and analysis system to enhance investigations into the effects of EP on tissue. The thermal effect can be monitored with an invasive and non-invasive technique and the first investigations showed reproducible results. Further investigations on the biological process of thermal necrosis due to EP is possible. A combination of spheroids and hydrogels could give a more complex and realistic in-vitro model where the limitations of each model alone can be overcome. Considering the possible reversible effect of EP, the established assays enable long-term investigation. An important improvement would be the upscaling of the hydrogel to increase image quality and also enable more fields of application. The system has the potential to be adapted to other ablation techniques due to the modular concept. Further studies could investigate different ablation parameters or techniques and the cellular response to the thermal influences.

## Data Availability

Data is provided within the manuscript. Original data can be provided upon request by contacting the corresponding author.
